# Underutilization of left heart catheterization in kidney transplant patients presenting with non-ST segment elevation myocardial infarction

**DOI:** 10.1016/j.ahjo.2023.100300

**Published:** 2023-05-04

**Authors:** Ahmad Mustafa, Samer Asmar, Chapman Wei, John Afif, Shahkar Khan, Taqi Rizvi, Radu Grovu, Mitchell Weinberg, Suzanne El-Sayegh

**Affiliations:** a Department of Internal Medicine, Staten Island University Hospital/Northwell Health, Staten Island, NY, USA; b Department of Cardiology, Staten Island University Hospital/Northwell Health, Staten Island, NY, USA; c Department of Nephrology, Staten Island University Hospital/Northwell Health, Staten Island, NY, USA

**Keywords:** Non-ST segment elevation myocardial infarction (NSTEMI), Kidney transplant (KT), Left heart catheterization (LHC)

## Abstract

**Background:**

Cardiovascular disease (CVD) is the leading cause of mortality in kidney transplant (KT) patients. The perceived risk of contrast-induced nephropathy (CIN) may create a reluctance to perform coronary angiography in patients presenting with non-ST segment elevation myocardial infarction (NSTEMI).

**Methods:**

National Inpatient Sample (NIS) Database was used to sample individuals presenting with NSTEMI. Patients were stratified into KT and Non-KT cohorts. Outcomes included left heart catheterization rates, mortality, arrhythmias, acute kidney injury/acute renal failure (AKI/ARF), and extended length of hospital stay (ELOS) (>72 h). Propensity matching (1:1 ratio) and regression analyses were performed.

**Results:**

Out of 336,354 patients with NSTEMI, 742 patients were in the KT group. KT patients were less likely to have LHC relative to non-KT patients (22.0 % vs 18.3 %); a difference that persisted on post-match analysis (27.1 % vs 19.4 %). On pre-match analysis, KT transplant patients that underwent LHC had lower mortality (10.3 % vs 0.7 %), AKI/ARF (44.6 % vs 27.9 %), arrhythmias (30.4 % vs 20.6 %) and lower ELOS (58.6 % vs 41.9 %). Post-match, KT cohort patient that underwent LHC had lower arrhythmias (OR:0.60[0.38–0.96]), AKI/ARF (OR = 0.51[0.34–0.77]), ELOS (OR:0.49[0.34–0.73]).

**Conclusion:**

KT patients underwent LHC much less frequently than their non-KT counterparts for NSTEMI. Coronary angiography and subsequent revascularization were associated with a significant decrease in morbidity and mortality. This theorized risk of CIN should not outweigh the benefit of LHC in KT patients.

## Highlights

• KT patients less likely to get coronary LHC after NSTEMI vs non-KT patients.
• Higher morbidity/mortality in KT patients that did not receive LHC after NSTEMI.
• Strategies to reduce bias against KT patients with NSTEMI are needed for optimal LHC rates.

## 1 Introduction

Kidney transplantation (KT) is the recommended treatment for end-stage renal disease (ESRD) patients and is associated with improved outcomes [1]. The survival benefit of kidney transplantation is mostly secondary to an overall decrease in cardiovascular disease (CVD) burden [2]. The incidence of acute myocardial infarction (AMI) in the ESRD population in the USA has been declining and is likely secondary to improvements in primary prevention efforts [3], [4].

Despite this risk reduction, KT patients remain at high risk for CVD-related morbidity and mortality, compared to general population [5], [6]. In fact, CVD is the leading cause of mortality in KT patients with a functioning allograft [7]. Specifically, AMI and its related complications remain the leading cause of death in this patient population [8], [9].

AMI encompasses ST-elevation myocardial infarction (STEMI) and non-STEMI (NSTEMI). The incidence of STEMI in KT patients is 1.3 times higher than that of the general population [3], [10]. At one year post-KT, the incidence of STEMI was 2.3 % and increased to 5.8 % in a 5-year follow-up period [10], [11], [12]. However, a history of KT is more likely to be associated with NSTEMI compared to STEMI upon presentation to the hospital [13]. Recent reports showed that in-hospital mortality among KT patients with STEMI is declining, while that of NSTEMI patients remains unchanged [13], [14].

KT patients are more prone to develop acute kidney injury (AKI) after receiving iodinated contrast based on recent emerging data [15], [16]. Despite the elevated risk of CVD in KT patients, this may create a reluctance to perform coronary angiography due to the perceived risk of contrast-induced nephropathy. Thus, this can create a dilemma of optimally treating coronary artery disease (CAD) while maximizing kidney graft integrity.

KT patients represent a unique and vulnerable patient population. Nationwide data evaluating the outcomes and complications of NSTEMI in KT patients are limited. A better understanding of these outcomes can provide valuable prognostication information that could enhance cardiovascular care for these patients while maximizing graft integrity. Hence, we sought to study the rates of left heart catheterizations (LHC) in KT vs non-KT transplant patients and its effect on outcomes and mortality.

## 2 Methods

Individuals presenting with NSTEMI were sampled from National Inpatient Sample (NIS) database (2016–2018). The NIS database is the largest inpatient database in the United States and is funded through Healthcare Cost and Utilization Project (HCUP) [17]. IRB is exempt as per the HCUP data use agreement since patient information is de-identified.

**Fig. 1:**
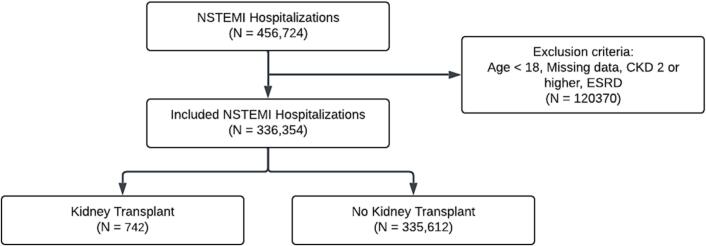
Schematics of study design. NSTEMI: non-ST segment elevation myocardial infarction, CKD: chronic kidney disease, ESRD: End stage renal disease.

Comorbidities and baseline demographics were collected using International Classification of Disease 10 codes (Supplementary Table 1). Patients were stratified into KT and non-KT groups. Patients with an age <18, those with missing data, or those with chronic kidney disease 2 or higher, including ESRD, were excluded from the study (Fig. 1).

The primary outcome was LHC rates between the KT and non-KT transplant cohorts. Secondary outcomes were analyzed in the KT group and compared patients who underwent LHC to those who did not. Secondary outcomes included in-hospital mortality, arrhythmias (a composite of atrial fibrillation, atrial flutter, supraventricular tachycardia, atrioventricular blocks, bundle branch blocks, ventricular tachycardia), AKI (including post-LHC AKI/acute renal failure), acute heart failure and extended length of hospital stay (ELOS; defined as >72 h).

KT and non-KT groups were then matched 1:1 using Greedy propensity with R version 4.1.2 (R Foundation for Statistical Computing, Vienna, Austria). The caliper for propensity score difference was 0.0000001. The two cohorts were matched on age, gender, race, CAD, carotid artery disease, chronic obstructive pulmonary disease, diabetes mellitus, dyslipidemia, hypertension, obesity, chronic heart failure, smoking, and peripheral vascular disease. After matching, the mean difference in propensity score was <0.1 which signified successful matching. A Chi-Square analysis was performed to confirm that the two groups were similar (p > 0.99).

Pre-match and post-match univariate analyses (chi-square and student's *t*-tests) were conducted for the primary outcome between the two cohorts. Continuous variables were analyzed using student t-tests and ANOVAs. Categorical variables were analyzed using chi-square analyses, and Fisher's exact tests. Only KT patients were included in the analysis for the secondary outcomes. Binary logistic regression analyses on pre-matched and post-matched KT cohorts were conducted. For the pre-matched cohorts, all the variables that were utilized for propensity score matching, and kidney transplant status were entered in the binary logistic regression model. For the post-match binary logistic regression model, only kidney transplant status was entered. A *p*-value <0.05 was considered statistically significant. All statistical analyses were done using IBM SPSS Statistics for Windows, version 28 (IBM Corp., Armonk, N.Y., USA) and R version 4.1.2 (R Foundation for Statistical Computing, Vienna, Austria).

## 3 Results

Out of a total of 456,724 patients, 336,354 patients were included in the study. There were 335,612 non-KT patients and 742 KT patients. KT patients were younger, Caucasians, and had a higher prevalence of CAD, diabetes mellitus, and peripheral vascular disease. The non-KT group had more females and more prevalence of chronic obstructive pulmonary disease, obesity, and smoking (Table 1; Fig. 1).

**Table 1:** Baseline characteristics and comorbidities. Values are N(%) or mean ± SD.

Comorbidities	Non-KT N = 335,612	KT N = 742	P-value
Age (years)	68.5 (13.8)	64.2 (10.5)	<0.001
Female	144,025 (42.9)	278 (37.5)	0.003
White	251,220 (74.9)	454 (61.2)	<0.001
Coronary artery disease	209,629 (62.5)	511 (68.9)	<0.001
Carotid artery disease	2167 (0.6)	3 (0.4)	0.642
Chronic obstructive pulmonary disease	75,918 (22.6)	70 (9.4)	<0.001
Diabetes mellitus	128,053 (38.2)	499 (67.3)	<0.001
Dyslipidemia	152,627 (45.5)	345 (46.5)	0.578
Hypertension	332,838 (99.2)	733 (98.8)	0.246
Obesity	61,826 (18.4)	106 (14.3)	0.004
Chronic Heart failure	102,514 (30.5)	241 (32.5)	0.253
Smoking	92,349 (27.5)	175 (23.6)	0.017
Peripheral vascular disease	35,623 (10.6)	109 (14.7)	<0.001

On pre-match analysis, there was a higher rate of LHC in the non-KT group compared to the KT group (22 % vs 18.3 %; p = 0.015) (Table 2). In the KT group, 136 patients underwent LHC, and 604 did not. Individuals who had LHC had lower in-hospital mortality (0.7 % vs. 10.3 %; p < 0.001), AKI/acute renal failure (28.7 % vs. 44.6 %; p < 0.001), arrhythmias (20.6 % vs. 30.4 %; p < 0.023), and lower rates of an ELOS (41.9 % vs. 58.6 %; p < 0.001). There was a trend towards reduced cardiac arrest (2.2 % vs. 3.5 %) and acute heart failure (21.3 % vs. 24.9 %); however, this did not reach statistical significance (Table 3).

**Table 2:** Primary Outcome- Rates of left heart catheterization between KT and non-KT cohorts before and after matching. Values are N(%). KT = kidney transplant.

	Unmatched			Matched		
	Non-KT (N = 335,612)	KT (N = 742)	P-value	Non-KT (N = 675)	KT-matched (N = 675)	P-value
Left heart catheterization	73,919 (22.0)	136 (18.3)	0.015	183 (27.1)	131 (19.4)	<0.001

**Table 3:** Univariate analysis for secondary outcomes within the kidney transplant group. LHC = Left heart catheterization.

	Unmatched			Matched		
	No LHC (N = 604)	LHC (N = 136)	P-value	No LHC (N = 544)	LHC (N = 131)	P-value
Mortality	62 (10.3)	1 (0.7)	<0.001	56 (10.3)	0 (0.0)	<0.001
Cardiac arrest	21 (3.5)	3 (2.2)	0.597	19 (3.5)	2 (1.5)	0.398
Acute kidney injury	270 (44.6)	38 (27.9)	<0.001	239 (43.9)	37 (28.2)	0.001
Acute heart failure	151 (24.9)	29 (21.3)	0.377	133 (24.4)	28 (21.5)	0.491
Arrhythmia	184 (30.4)	28 (20.6)	0.023	165 (30.3)	192 (20.8)	0.031
Extended length of hospital stay (ELOS)	355 (58.6)	57 (41.9)	<0.001	313 (57.4)	52 (40.0)	<0.001

**Fig. 2:**
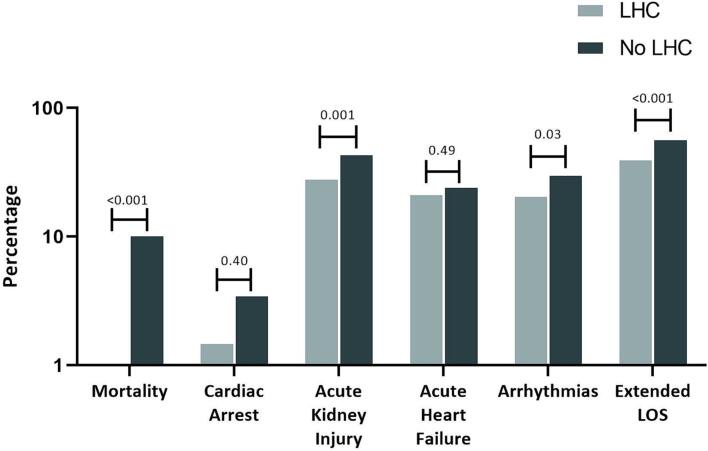
Post-match analysis outcomes of kidney transplant patients who underwent left heart catheterization (LHC) versus those who did not.

After 1:1 matching, the LHC rate was still higher in the non-KT group (27.1 % vs. 19.4 %; p < 0.001) (Table 2). KT patients that underwent LHC still had lower rates of AKI/acute renal failure, arrhythmia, and ELOS. Lastly, post-match, no KT patients that underwent LHC had in-hospital mortality (Table 3; Fig. 2).

KT patients who underwent LHC and those who did not were compared for secondary outcomes. Table 4 shows the differences in baseline characteristics. Patients who underwent LHC had a higher prevalence of CAD and dyslipidemia, and lower rates of chronic heart failure. Binary logistic regression analysis for secondary outcomes revealed similar results pre-match and post-match (Table 5). Patients that underwent LHC had lower rates of arrhythmia [OR: 0.60(0.38–0.96); p = 0.03] and extended LOS [OR: 0.49(0.33–0.73); p < 0.001].

**Table 4:** Baseline Characteristics and comorbidities of Kidney Transplant cohort. Values are N(%) or mean ± SD. LHC = Left heart catheterization.

Comorbidities	LHC N = 136	No LHC N = 606	P-value
Age (years)	63.1 ± 10.6	64.5 ± 10.4	0.161
Female	50 (36.8)	228 (37.6)	0.852
White	88 (64.7)	366 (60.4)	0.351
Coronary artery disease	109 (80.1)	402 (66.3)	0.002
Carotid artery disease	0 (0.0)	3 (0.5)	1.000
Chronic obstructive pulmonary disease	10 (7.4)	70 (9.3)	0.358
Diabetes mellitus	92 (67.6)	407 (67.2)	0.913
Dyslipidemia	76 (55.9)	269 (44.4)	0.015
Hypertension	136 (100.0)	597 (98.5)	0.378
Obesity	20 (14.7)	86 (14.2)	0.877
Chronic Heart failure	29 (21.3)	212 (35.0)	0.002
Smoking	32 (23.5)	143 (23.6)	0.987
Peripheral vascular disease	17 (12.5)	92 (15.2)	0.425

**Table 5:** Binary logistic regression analysis for secondary outcomes within the kidney transplant group.

	Unmatched			Matched		
	Adjusted OR	CI	P-value	Adjusted OR	CI	P-value
Mortality	0.079	0.011–0.576	0.012	NA		
Acute kidney injury	0.508	0.333–0.775	0.003	0.509	0.336–0.773	0.002
Arrhythmia	0.590	0.370–0.940	0.26	0.604	0.381–0.958	0.032
Extended length of hospital stay (ELOS)	0.530	0.359–0.783	0.001	0.494	0.335–0.730	<0.001

One non-KT patient had a post-LHC AKI/ARF, whereas no KT patients had post-LHC acute kidney injury/acute renal failure.

## 4 Discussion

In this study, KT patients with NSTEMI were less likely to undergo LHC than their non-KT counterparts. LHC in KT patients presenting with NSTEMI was associated with less AKI, arrhythmias, less ELOS, and lower mortality.

CVD remains the leading cause of morbidity and mortality in KT patients [18]. Over 10 % of KT patients experience an AMI within three years of transplantation [19]. Traditional CAD risk factors such as diabetes mellitus and hypertension, are known to be more prevalent in KT patients. These factors are often the underlying etiologies of ESRD and further lead to adverse cardiovascular events [20]. Previous studies also suggested that cardioprotective medications are either less effective or underutilized in KT individuals [20], [21].

Previous cohort studies have evaluated the cardiovascular outcomes following KT. A 5-year follow-up study of KT recipients reported that 5.8 % of the KT group and 2.8 % of the non-KT group developed MI. Similar to our results, patients in the KT group had higher baseline comorbidities. It was concluded that KT was independently associated with a 45 % higher risk of MI when compared to non-KT, with a predominance of NSTEMI. They showed that MI in KT patients was independently associated with a 15 % increased risk of mortality for patients with MI and non-KT [12]. This study provided valuable trends but did not explore whether patients received an intervention or medical management.

Recent literature has focused on examining disparities in procedures based on age, sex, and race. Mcneil et al. coined the term “renalisim” to reflect the prejudice of coronary angiography underutilization after AMI in elderly patients with CKD compared to elderly patients without CKD [22]. Studies have also examined the variation in the rates of cardiac procedures due to the presence of certain coexisting medical conditions [23], [24]. Certain coexisting conditions frequently resulted in conservative medical management, perhaps secondary to physicians' aversion to risk [22]. Being a kidney transplant recipient is one of those conditions. In the study by Fox et al., KT patients were less likely to undergo coronary angiography or revascularization after AMI [21]. This is likely due to the fear of inducing nephrotoxicity by radiocontrast media. Management of KT population requires a non-biased multidisciplinary approach with careful balancing of the potential benefit of coronary angiography with the risks of radiocontrast-associated nephropathy vs a more conservative medical approach.

Transplanted kidneys can be more susceptible to hemodynamic insults that lead to AKI. The absence of sympathetic innervation predisposes it to hyperfiltration injury [25]. Another predisposing factor is the concomitant use of calcineurin inhibitors, which have nephrotoxic effects. These medications may cause afferent arteriolar vasoconstriction and tubular insults [26]. Our study showed that KT patients who underwent LHC had paradoxically lower rates of AKI/acute renal failure. The likely explanation for this finding may be multifactorial. Management of hospitalized KT patients entails early consultation with transplant nephrologists that contributes to improved outcomes [27], [28]. This early collegial and collaborative management between nephrologists and cardiologists may lower the risk of cardio-renal syndromes and leads to optimization of fluid-dynamic management, hence reducing the risk of AKI in this cohort [29].

Our study has certain limitations. It is a retrospective design that may have led to certain selection biases. Thrombolysis in Myocardial Infarction (TIMI) and Global Registry of Acute Coronary Events (GRACE) score, commonly used to guide management in NSTEMI patients, could not be calculated. However, these limitations were largely eliminated by using propensity-score matching. The data's accuracy depends on coding practices that may vary between hospitals. However, the large size of the database reduces this potential limitation. NIS is an administrative database and lacks information on medication utilization and outpatient follow-up data. Data on functional status of KT patients was not available which might have influenced the treatment strategy. Propensity scores can only adjust for associations among observed covariates and the chosen treatment strategy but cannot discern what specific medications were utilized. Thus, there can be residual unmeasured confounders. Outcomes in the NIS are limited to index hospitalizations and hence only short-term outcomes could be analyzed.

## 5 Conclusion

The results of our study confirmed that KT patients were less likely to receive coronary angiography after NSTEMI compared to those without KT. This significant difference persisted even after 1:1 matching for baseline demographics and comorbidities. Coronary angiography and subsequent revascularization were associated with a significant decrease in morbidity and mortality in this population. Conventionally, the theoretical risk of nephrotoxicity secondary to cardiac angiography is one of the reasons this patient population received fewer coronary interventions. Strategies designed to decrease KT bias and allow KT patients to undergo coronary angiography for NSTEMI should be further explored as the benefit could outweigh the potential risks.

The following is the supplementary data related to this article.

Supplementary Table 1 ICD-10 codes. ESRD- End-stage renal disease. Supplementary Table 1

## Ethical statement

The study received proper ethical oversight and IRB was exempt as per HCUP DUA. The study did not receive any funding, and the authors have nothing to disclose.

## Funding

No external funding or financial support was received for this work.

## Declaration of competing interest

The authors declare that they have no known competing financial interests or personal relationships that could have appeared to influence the work reported in this paper.
