# Supplementary material for: Underutilization of left heart catheterization in kidney transplant patients presenting with non-ST segment elevation myocardial infarction
Source: Am Heart J Plus. 2023 May 4;30:100300. doi: 10.1016/j.ahjo.2023.100300 (PMC10946038; doi:10.1016/j.ahjo.2023.100300)
Supplement: Supplementary Table 1 — ICD-10 codes. ESRD- End-stage renal disease. [file mmc1.docx]

| **Comorbidities** | **ICD-10-DM** |
| --- | --- |
| Left heart catheterization | 02704ZZ, 02704Z6, 02704TZ, 02704T6, 02704FZ, 02704F6, 02704GZ, 02704G6, 02704EZ, 02704E6, 02704DZ, 02704D6, 027047Z, 0270476, 027046Z, 0270466, 027045Z, 0270456, 027044Z, 0270446, 02703ZZ, 02703Z6, 02703TZ, 02703T6, 03703FZ, 02703F6, 02703GZ, 02703G6, 02703EZ, 02703E6, 02703DZ, 02703D6, 0270376, 027036Z, 0270366, 027035Z, 0270356, 027034Z, 0270346 |
| Kidney transplant status | Z94.0 |
| Chronic kidney disease (Stage II to V, and ESRD/hemodialysis-dependent) | N18.2 – N18.5, Z49, N18.6, Z99.2 |
| Coronary artery disease | I25.10, I25.2, I25.8, I25.9 |
| Carotid artery disease | I65.2 |
| Chronic obstructive pulmonary disorder | J41-J44 |
| Diabetes mellitus Type 2 | E08-E13, O24 |
| Dyslipidemia | E78.0-E78.5 |
| Hypertension | I10-I13, I15-I16, I67.4, H3503, O10-O13, O16 |
| Obesity | E66.3, E66.8, E66.9, E66.01, E66.2 |
| Chronic heart failure | I11.0, I13.0, I09.81, I50.1, I50.20, I50.22, I50.30, I50.32, I50.40, I5.042, I50.810, I50.812, I50.814, I50.82, I50.83, I50.84, I50.89, I50.9, I51.81, I97.130, Z95.812 |
| Smoking history | Z87.891, F17.200 |
| Peripheral vascular disease | I70 |
| Cardiac arrest | I46.2, I46.8, I46.9, I97.121 |
| Acute kidney injury (including post-procedural acute kidney injury) | N99.0, N17 |
| Acute heart failure | I50.21, I50.23, I50.31, I50.33, I50.41, I50.43, I50.811, I50.813 |
| Arrhythmias (includes atrial fibrillation, atrial flutter, supraventricular tachycardia, AV nodal blocks, bundle branch blocks, ventricular tachycardia) | I44, I45, I48, I47.1, I47.20, I47.29 |

**Supplementary Table 1.** ICD-10 codes. ESRD- End-stage renal disease.
